# Experimental and Numerical Investigations on the Influences of Target Porosity and w/c Ratio on Strength and Permeability of Pervious Concrete

**DOI:** 10.3390/ma18173951

**Published:** 2025-08-22

**Authors:** Fei Liu, Zhe Li, Bowen Liu, Zhuohui Yu, Zetong Li, Mengyuan Zhu, Yanjie Wang, Xizhou Ding

**Affiliations:** 1 School of Civil and Transportation Engineering, Beijing University of Civil Engineering and Architecture, Beijing 102616, China; liufei@bucea.edu.cn (F.L.); lizhe725@163.com (Z.L.); liubowen0502@163.com (B.L.); dxz15698386546@163.com (X.D.); 2 School of Economics and Management, Jilin Jianzhu University, Changchun 130118, China; m15543127938@163.com (Z.Y.); m13137308868@163.com (Z.L.); 3 School of Engineering, Design and Built Environment, Western Sydney University, Kingswood, NSW 2751, Australia; 4 School of Civil Engineering, Hebei University of Engineering, Handan 056038, China; wangyanjie@hebeu.edu.cn

**Keywords:** pervious concrete, steel slag aggregate, compressive strength, permeability, porosity

## Abstract

Pervious concrete is a promising sustainable pavement material for sponge city construction. The incorporation of Steel Slag Aggregate (SSA) as a substitute for natural aggregates has the double role of clean production with significant economic and environmental benefits. While the strength and permeability, known as two critical design parameters of pervious concrete, are closely linked to its porosity, there is limited research on the influence of the porosity on the mechanical properties of pervious concrete. In this paper, both experimental and numerical investigations were performed, focusing on the influence of target porosity on the strength and permeability of pervious concrete with and without SSA. Three different target porosities (15%, 20%, and 25%), five distinct water-to-cement (w/c) ratios (0.25, 0.28, 0.30, 0.33, and 0.35), and five SSA replacement ratios (0, 25%, 50%, 75%, and 100%) were considered in this study. A two-dimensional (2D) finite-element (FE) model was developed, with which the failure mode and the strength variation of pervious concrete under different target porosities were analyzed and verified with the experimental results. The results showed that the porosity had a significant influence on both the strength and permeability of pervious concrete, while the influence of the w/c ratio is marginal. There existed an optimal w/c ratio of 0.3, for which pervious concrete with porosities of 15%, 20%, and 25% achieved 28-day compressive strengths of 27.8, 20.6, and 15.6 MPa and permeability coefficients of 0.32, 0.58, and 1.02 cm/s, respectively. Specifically, at the lowest porosity of 15%, the replacement of 100% SSA resulted in the largest improvement in the compressive strength up to 37.86%. Based on the regression analysis, a series of empirical equations correlating the porosity, strength and permeability of pervious concrete was formulated and validated against the experimental data. The findings presented herein are expected to provide references to the practical evaluation of the optimal mix proportion of previous concrete, considering specific and demanding engineering requirements.

## 1. Introduction

Continuous expansion of road networks, acceleration of industrialization, and rapid development of urbanization have led to global concerns, including a shortage of natural aggregates, accumulation of industrial by-products, and urban environmental problems represented by the heat island effect. Within this context, pervious concrete—a sustainable and porous building material—has gained considerable attraction in the last two decades [1,2,3,4,5,6]. The intrinsic void structure characteristic of pervious concrete facilitates rapid infiltration of rainfall, consequently mitigating surface runoff and substantially reducing the risk of urban flooding. Furthermore, the interconnected porosity of this material promotes the evaporation of soil moisture, thereby aiding in surface temperature regulation and ameliorating the urban heat island effect. Additionally, pervious concrete contributes to enhanced urban livability by attenuating noise pollution.

Pervious concrete typically exhibits a porosity of 15–30% to ensure adequate water permeability. However, this inherent void structure invariably results in reduced mechanical properties compared to conventional concrete, with typical compressive strengths ranging from 7 to 25 MPa [7,8]. The strength of pervious concrete is governed by the aggregate strength, the cement matrix strength, and the bond strength at the aggregate-to-cement interface. This performance limitation has historically constrained its broader application, thereby stimulating extensive research focused on enhancing its structural integrity. Many studies consistently indicate that aggregates exert a predominant influence on the performance of pervious concrete [9,10,11]. Specifically, the size and distribution of aggregate particles directly dictate pore characteristics (size and volume fraction) and the pattern of aggregate interlocking, all of which critically influence the compressive strength. For instance, employing larger aggregates tends to create larger pores, potentially compromising the concrete properties, whereas optimizing aggregate gradation can refine the pore network and therefore improve strengths. Furthermore, utilizing aggregates possessing higher inherent strength enhances the material’s load-bearing capacity and overall mechanical robustness [12,13,14].

Steel slag, an industrial by-product of steelmaking, was historically classified as waste. Its principal chemical constituents include oxides of iron, magnesium, aluminum, and calcium. Motivated by the need to mitigate the environmental and logistical challenges posed by steel slag accumulation, researchers have increasingly focused on its recycling potential. Notably, certain studies have demonstrated that steel slag exhibits specific gravity and compressive strength properties slightly superior to those of basalt [15]. Consequently, the incorporation of Steel Slag Aggregates (SSA) into pervious concrete presents a multi-faceted opportunity, addressing both material recycling and performance enhancement. Investigations into SSA pervious concrete have primarily centered on its mechanical and environmental performance. It is indicated that concrete incorporating SSA typically achieves superior mechanical strength and a higher permeability coefficient compared to its natural aggregate (NA) counterparts [16,17]. Reported compressive strengths generally exceed 10 MPa, while porosities often surpass 15%, though these values are contingent upon factors such as cement type, slag source, curing conditions, and aggregate gradation [18,19,20]. This promising performance has facilitated tentative applications, notably in sidewalk construction. Also, SSA pervious concrete has been shown to possess notable capabilities for CO_2_ absorption and low-frequency noise attenuation [21], thereby contributing positively to the objectives of sponge city development [22]. Nevertheless, the full potential of SSA pervious concrete remains underexplored. A significant gap exists in the current literature, as most studies have concentrated on macroscopic laboratory assessments; the performance-strengthening mechanisms induced by SSA are relatively limited. Moreover, there is a lack of research examining the meso-structural characteristics and the behavior of the aggregate-cement interface transition zone (ITZ) under the influence of SSA [23]. Addressing these knowledge gaps is crucial for achieving substantial performance improvements and broadening the practical application scope of SSA pervious concrete.

It has been recognized that excessive porosity may result in serious reductions in the strength of pervious concrete. Recent research has extensively investigated the relationship between porosity and water permeability in pervious concrete. For instance, Akand et al. [24] employed two-dimensional image analysis coupled with finite-element modelling to assess the influence of void distribution on permeability. Pieralisi et al. [25] developed a discrete-element computational fluid dynamics model for the theoretical exploration of water flow through pervious concrete. Empirical studies by Elango and Revathi [26] proposed a strong correlation between porosity and permeability metrics. Li et al. [27] conducted comprehensive testing on 24 mixtures of pervious concrete with varying water-cement (w/c) ratios (0.20–0.35) and porosity values (15–40%). It was shown that both the w/c ratio and the paste volume critically influence the resultant porosity, permeability, and mechanical strength of pervious concrete. A notable inconsistency exists across studies regarding the methodologies employed to measure water permeability. Yang and Jiang [28] utilized a falling-head permeability test, contrasting with the constant head method adopted by Pieralisi et al. [25].

From a practical design purpose, the mix proportions of pervious concrete must be carefully tailored to meet specific permeability requirements, as these needs vary across different regions and applications. Among the design parameters, the target porosity is particularly critical as it directly influences the permeability characteristics of pervious concrete [29,30]. Earlier findings indicated that the effect of certain fiber materials on the mechanical performance of pervious concrete is minimal compared to their impact on porosity [31,32]. A higher target porosity correlates with better permeability but may compromise the strength [33,34,35,36]. Despite this, research specifically focused on the mechanical properties of pervious concrete under different target porosities remains relatively scarce. The quantitative relationship between the fundamental parameters, like compressive strength and permeability coefficient and the target porosity has not been established. Further research is essential to consider these factors, optimize material proportions and production techniques, and achieve the best possible compromise between strength and permeability of pervious concrete.

The literature review confirms that porosity level and w/c ratio are key determinants of pervious concrete’s mechanical performance. However, research on the impact of target porosity on its mechanical properties and failure behavior of pervious concrete remains limited. The objective of the present study is to perform experimental and numerical investigations to evaluate the influence of target porosity on the mechanical behavior of pervious concrete with and without SSA. Three different target porosities (15%, 20%, and 25%) and five distinct water-to-cement (w/c) ratios (0.25, 0.28, 0.30, 0.33, and 0.35) were considered, and their effects on the failure mode of pervious concrete were elaborated. Finally, an empirical model linking the strength and permeability was presented and further verified with the self-conducted experimental results.

## 2. Experimental Investigation

The study consisted of two parts: experimental investigation and numerical modeling. Guidelines such as ACI PRC-522-23 commonly propose an experimental method for mixture proportion, focused on determining the optimal paste volume required to adequately coat the aggregate particles [37]. This experimental approach entails the formulation of trial mixes, followed by iterative adjustments to ensure the resultant pervious concrete meets the targeted hydraulic and mechanical performance criteria.

### 2.1. Raw Materials

The pervious concrete examined in this study was fabricated using a fundamental set of raw materials, namely coarse aggregates, cement, mineral admixtures, chemical admixtures, and water. The coarse aggregates used included crushed granite rock and steel slag. The crushed granite has a peak size of 10 mm, apparent density of 2870 kg/m^3^, bulk density of 1550 kg/m^3^, clay content of 0.4%, needle and flake content of 2.2%, compacted bulk porosity of 46%, and crush value of 6.4%. Its particle size distribution (PSD) as characterized through sieve analysis, which indicated that 2% of the particles were finer than 5 mm (passing the 5 mm sieve), while 98% resided within the 5–10 mm range (passing the 10 mm sieve but retained on the 5 mm sieve). The steel slag aggregate (SSA) has a size of 5 to 10 mm, an apparent density of 3290 kg/m^3^, a compacted bulk density of 1870 kg/m^3^, a compacted bulk porosity of 0.4%, and a crush value of 6.3%. The results are summarized in Table 1. The application of steel slag necessitated a prolonged storage and aging phase to ensure its stability and prevent detrimental volume expansion within the concrete matrix, a phenomenon often linked to the presence of free calcium oxide. The chemical components of steel slag are shown in Table 2.

**Figure 1:**
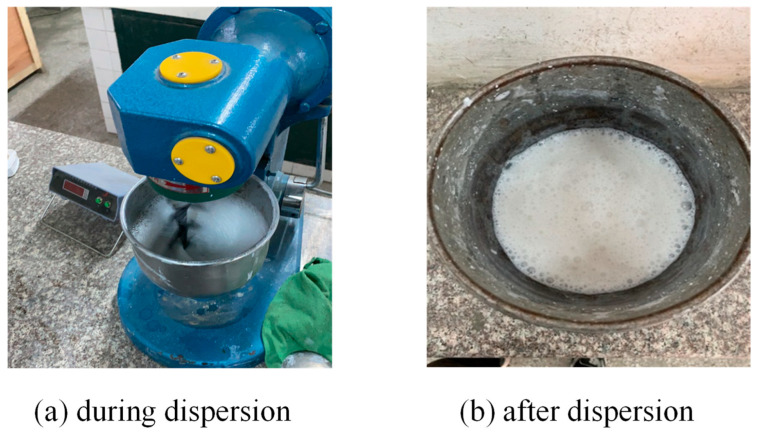
Preparation of nano-silica.

**Figure 2:**
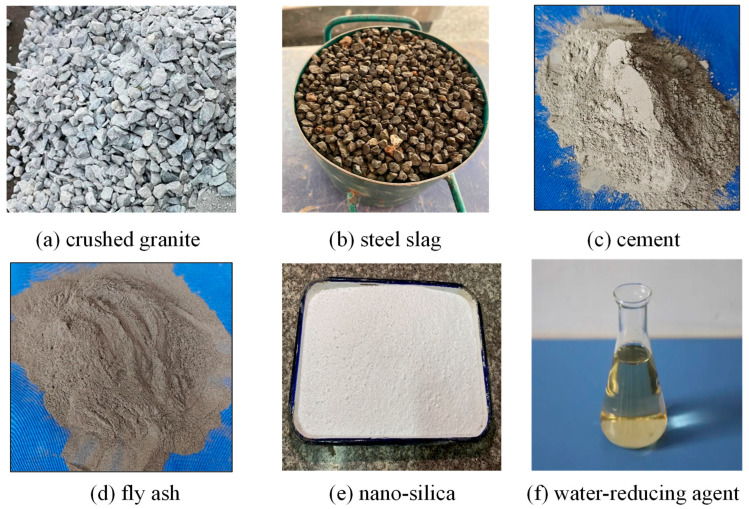
Raw materials.

As for the cementitious matrix, the P.O. 42.5 grade ordinary Portland cement meeting the Chinese standard GB 175-2007 [38] and the grade II fly ash following the Chinese standard “Technical code for application of fly ash concrete” (GB/T50146-2014 [39]) were utilized. The cement had a density of 3.2 g/cm^3^, specific surface area of 335 m^2^/kg, 3 d and 28 d flexural strengths of 5.8 and 7.7 MPa, and 3 d and 28 d compressive strengths of 30.1 and 53.2 MPa, respectively. The fly ash has a fineness of 26.3, density of 2.52 g/cm^3^, water demand ratio of 96%, ignition loss of 5.68%, and moisture content of 0.1%. To enhance the fluidity characteristics of the mixture, a polycarboxylate acid superplasticizer exhibiting a water reduction rate exceeding 30% was incorporated, with its main parameters given in Table 3. Additionally, the testing procedure utilized standard municipal tap water as the water source. The incorporation of a suitably proportioned nano-silica additive was found to augment the paste strength and refine the interfacial transition zone (ITZ) at the aggregate-mortar interface. This, in turn, leads to an improvement in the overall mechanical properties of the pervious concrete. For the purpose of dispersion, a conventional high-speed mixing technique was employed within the mixer to distribute the nano-silica particles throughout the cementitious matrix, as depicted in Figure 1. The raw materials used for preparing the pervious concrete with and without steel slag are summarized in Figure 2.

### 2.2. Mix Design

Mix design for pervious concrete needs to simultaneously satisfy both strength and permeability requirements. To investigate the effects of porosity and water-to-cement (w/c) ratio on the properties of pervious concrete, three levels of porosity (15%, 20%, and 25%) and five different w/c ratios (0.25, 0.28, 0.30, 0.33, and 0.35) were considered for the preparation of the pervious concrete (PC). The influence of the SSA was evaluated through the use of five levels of SSA replacement ratio (0%, 25%, 50%, 75%, and 100%) for the preparation of the Steel Lag Pervious Concrete (SSPC). No fine aggregate was used for both PC and SSPC.

The mix design method for SSPC differs significantly from that of ordinary concrete. During the proportioning process, it is critical to ensure that the cement slurry only coats the surface of the steel slag aggregates, without filling the interstitial spaces between them. This approach facilitates the formation of an effective, interconnected pore structure, which is essential for meeting the porosity and permeability requirements of pervious concrete. In this study, the volume method was employed to design the mix proportion of SSPC. The fundamental principle of this method assumes that within a specified volume, the steel slag aggregates are densely packed, with their surfaces uniformly coated and bonded by the slurry. This results in a framework-pore structure upon hardening. By subtracting the volumes of aggregates and slurry from the total volume, the remaining space represents the total pore volume, from which the specimen’s porosity is derived.

Herein, assuming a fixed volume of 1 m^3^, the unit volume dosages of aggregate, cement, water, and additives are denoted as *μ_s_*, *μ_c_*, *μ_w_*, and *μ_a_*, respectively. Their corresponding apparent densities are represented by *ρ_i_* (*i* = *s*, *c*, *w*, and *a*). Assuming that the target porosity is *P*, the relationship between the aforementioned parameters can be established using the following equation:

(1) $$\frac{\mu_{s}}{\rho_{s}}+\frac{\mu_{c}}{\rho_{c}}+\frac{\mu_{w}}{\rho_{w}}+\frac{\mu_{a}}{\rho_{a}}+P=1$$

The National Ready Mixed Concrete Association (NRMCA) states that the typical porosity of pervious concrete ranges from 15% to 25% [40]. Additionally, pervious concrete generally employs a relatively low w/c ratio, which has been observed to vary between 0.28 and 0.40 in previous investigations. Consequently, the water-cement ratio for the SSPC prepared in this study was set at 0.30, while the target porosity was established at 15%, 20%, and 25%. This selection aligns with the general industry standards and ensures the material meets both strength and permeability requirements. The formulation of PC and SSPC mixtures follows a systematic procedure outlined as follows:

(i) *Target Porosity and Aggregate Preparation*. Specify the desired porosity, choosing a target value such as 15%, 20%, or 25%. Next, prepare the coarse aggregates (including crushed granite and steel slag, SS) according to established grading curves. The mass of these coarse aggregates required per cubic meter is initially calculated based on their close-packing density. To account for practical construction considerations, this calculated mass is then reduced by applying a factor of 0.98.
(ii) *Void Content Assessment*. Determine the void volume inherent within the densely packed coarse aggregate. In the close-packed state, the aggregate particles in pervious concrete are envisioned to be uniformly coated with cement paste. Upon solidification, this process facilitates the formation of the desired porous structure, where the interstitial voids present in the packed state evolve into the interconnected pores within the hardened concrete.
(iii) *Cement and Water Calculation*. Establish an initial water-to-cement (w/c) ratio for the intended mixture, selecting from suitable values such as 0.25, 0.28, 0.30, 0.33, and 0.35. Based on this ratio, compute the required quantities of cement and water. Given that the mixture design targets a specific porosity, these quantities can be determined using the following derivations. The determination of aggregate content, paste volume, water requirement, and admixture dosage is governed by Equation (1). Table 4 and Table 5 provide the detailed mixing ratios for the PCs and SSPCs, where the w/c ratio for the SSPC specimens is 0.30. Table 6 presents the physical and mechanical properties of the mixed aggregate using crushed granite and steel lag.

### 2.3. Specimen Preparation

A suitable molding method is essential to avoid slurry settlement and pore blockage in pervious concrete, ensuring the aggregate particles can form a proper framework-pore structure. All pervious concrete mixtures were prepared based on the cement paste encapsulation method. First, pre-weighed aggregates were loaded into the mixer. Then, a portion of the total mixing water was added to dampen the aggregate surfaces, followed by a 30-s mixing cycle to begin blending. Subsequently, the binder materials were introduced and mixed for an additional 30 s. Finally, the mixing water, along with any admixtures (e.g., superplasticizer), was incorporated, followed by a concluding mixing period of 1 min to ensure a homogeneous pervious concrete mixture.

**Figure 3:**
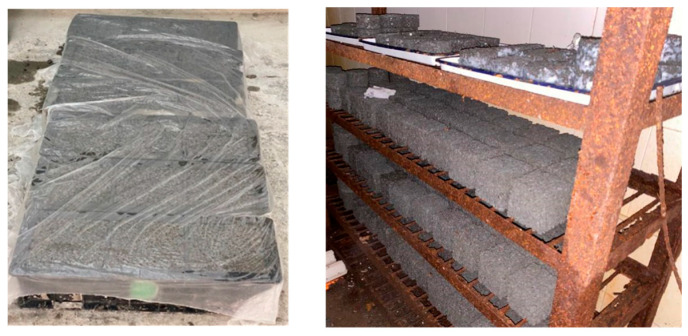
Curing of PP and SSPC specimens.

To preserve the internal void network essential for permeability, specimens were cast without mechanical vibration. Immediately after mixing, the fresh concrete was placed into the molds in three equal layers (each comprising one-third of the mold’s volume). Each layer was hand-compacted by inserting a standard steel rod approximately 25 times, moving from the edges toward the center. This technique ensured uniform compaction while carefully avoiding damage to the void structure. Specimens were made in two sizes: 100 mm × 100 mm × 100 mm cubes for compressive strength tests, and Φ 100 mm × 100 mm cylinders for permeability assessment. After casting, the specimens remained in their molds for 24 h at room temperature. They were then demolded and cured under standard conditions (20 ± 2 °C, 95% relative humidity) until testing at 28 days, as shown in Figure 3.

**Figure 4:**
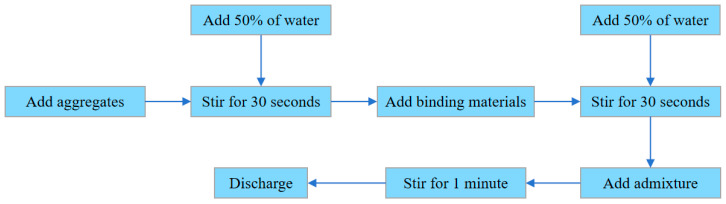
Preparation of the previous concrete.

To achieve the desired w/c ratios, polycarboxylic acid superplasticizer was used as a water-reducing agent. For the purpose of ensuring consistent and satisfactory workability across PC mixtures prepared with different w/c ratios, trial batching experiments were conducted alongside cement paste flow tests. Three w/c ratios, 0.25, 0.30, and 0.35, were selected, while keeping the target porosity at 20%. It was found that dispersion occurred in the mixtures with w/c ratios of 0.25 and 0.30, while this tendency decreased significantly when the w/c ratio reached 0.35, leading to notably improved cohesion and excellent adhesion between the cement paste and aggregates. Figure 4 shows the preparation process of the PC specimens.

In the present study, the selection of the sample geometry for determining the elastic modulus, the compressive strength, P-wave velocity, and permeability coefficient of pervious concrete was meticulously undertaken in accordance with the pertinent design guidelines. The elastic modulus and compressive strength tests were performed in accordance with ASTM C469 [41] and GB/T 50081-2019 [42], respectively. Similarly, the P-wave velocity and permeability coefficient tests were carried out in line with the procedures specified in ASTM C597/C597M-16 [43] and CJJ/T 135-2009 [44], respectively. In these tests, three target porosities (15, 20, and 25%) and five w/c ratios (0.25, 0.28, 0.30, 0.33, and 0.35) were considered, with three replicate specimens in each group. In compliance with these existing designations, cubic specimens with 100 × 100 × 100 mm^3^ were cast for the compressive tests of pervious concrete, accounting for a total of 45 specimens. Analogously, cylindrical specimens with dimensions of Φ100 × 200 mm were fabricated to determine the elastic modulus, again amounting to a total of 45 specimens. For the P-wave velocity test and permeability test, prismatic specimens with dimensions of 150 × 150 × 530 mm^3^ and cylindrical specimens with dimensions of Φ100 × 200 mm were prepared, summing up to 90 specimens in total. A recent investigation [45] has indicated the presence of a notable size effect in pervious concrete. Therefore, it is desirable to explore the influence of specimen geometry on the mechanical properties of pervious concrete. This will be the focus of our future studies.

### 2.4. Test Methods

#### 2.4.1. Strength Tests

In order for the evidence to be reliable, the elastic modulus of pervious concrete should be determined on the samples before the compressive strength test. The elastic modulus tests were conducted on cylindrical specimens with dimensions of Φ 100 × 200 mm [46,47]. During the tests, a total of forty-five test specimens were tested using the universal testing machine (UTM) with a capacity of 2000 kN at a loading rate of 0.5 MPa/s. The elastic modulus of the concrete was evaluated in accordance with ASTM C469 [41], wherein axial deformation of cylindrical specimens under compression was measured via a Linear Variable Displacement Transducer (LVDT) (manufactured by Liyang Instrument Factory, Liyang, China) positioned at mid-height. The elastic modulus was computed as the average slope of the ascending loading segments of the stress-strain curve across three loading/unloading cycles, each loaded to 40% of the ultimate compressive strength.

**Figure 5:**
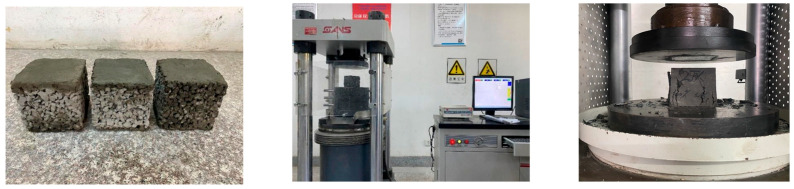
Strength tests.

The compressive strengths of PC and SSPC were evaluated after 28 days of standard curing, following the procedures outlined in the Chinese standard GB/T 50081-2019 [42]. Compressive strength tests were performed on cubic specimens with 100 mm × 100 mm × 100 mm. Before testing, caps were applied to the ends of the specimens. The choice of capping material depended on the surface condition of the concrete samples. For conventional concrete with smooth top and bottom surfaces, rubber caps were typically used. However, sulfur caps were applied to specimens with rough surfaces, such as pervious concrete. Since sulfur capping can significantly increase the compressive strength of pervious concrete by effectively restraining the aggregates at the top surface, it was used for all specimens in this study. A hydraulic universal testing machine (Type SYE-2000BS, manufactured by Guangzhou Boshun Experimental Instruments Co., Ltd., Guangzhou, China) was employed, applying a constant loading rate of 0.5 MPa/s during the test, as shown in Figure 5. The measured strength values for each test condition were calculated as the average of the test results from three individual specimens.

#### 2.4.2. Permeability Tests

**Figure 6:**
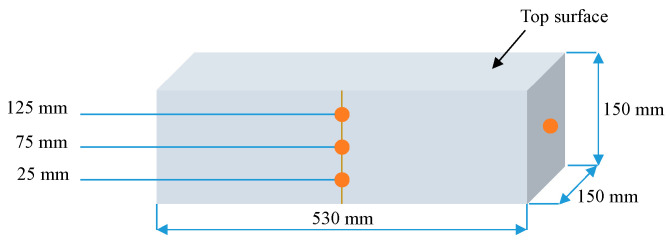
Face-to-face measurement method of ultrasonic wave velocity test.

The P-wave velocity should be determined on the samples before exposure to permeability tests. Figure 6 shows the schematic diagram of the measurement method of the ultrasonic wave velocity test. The wave velocity was determined via the face-to-face method at 28 days and calculated using the following equation [48].

(2) $$v=\frac{l}{t}$$

where *v* (m/s) represents the ultrasonic velocity; *l* (m) is the distance between oscillators, equal to the specimen length; and *t* (s) denotes the ultrasonic propagation time.

For the face-to-face method measurements (Figure 6), a pair of ultrasonic oscillators (manufactured by Beijing Zhibolian Technology Co., Ltd., Beijing, China.) with 50 mm in diameter and 50 kHz in frequency was used on a prismatic specimen (150 × 150 × 530 mm^3^). Two sets of measurements were performed. For the end-to-end path, the oscillators were placed on both end surfaces. For the mid-span path, they were positioned on side surfaces at 25, 75, and 125 mm from the bottom. At each measurement position, the propagation time was recorded five times to ensure repeatability.

The assessment of pervious concrete permeability, conventionally expressed as a rate in millimeters per second (mm/s), necessitates special consideration. Standardized testing protocols designed for conventional dense concrete are generally inadequate for pervious concrete specimens due to their significantly higher permeability. Herein, the permeability coefficient of PC and SSPC specimens was determined with the constant head (CH) permeameter method, directly applying Darcy’s law based on the Chinese standard CJJ/T 135-2009 [44].

**Figure 7:**
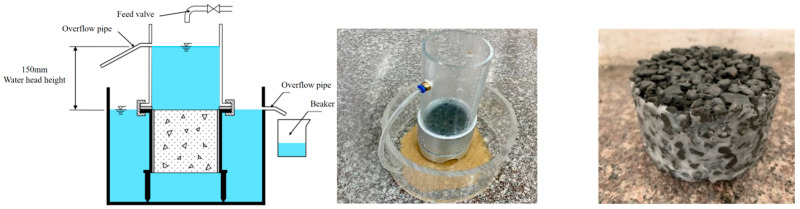
Permeability tests.

After the 28-day curing period, specimens were uniformly coated with petroleum jelly on their lateral surfaces to ensure effective lateral sealing. The sealed specimens were then placed in a permeameter, and the integrity of the seal was verified. Once the satisfactory sealing was confirmed, the entire setup was submerged in a water tank. Water was added incrementally from above, maintaining a constant hydraulic head of 20 cm. The test commenced only after a stable flow was observed at the overflow outlet. The volume of water (*Q*) discharged over a 5-min interval was collected using a graduated cylinder and recorded. This measurement was repeated three times to obtain an average value. The hydraulic head (*H*) within the permeameter was measured precisely to the nearest 1.0 mm, and the water temperature (*T*) in the overflow tank was recorded using a thermometer with a precision of 0.5 °C. The experimental setup and specimens are depicted in Figure 7. The average permeability coefficient was calculated using the following equation:

(3) $$K_{T}=\frac{Q\times L}{A\times H\times t}$$

where $K_{T}$ is the permeability coefficient at the test water temperature of *T* (cm/s), *A* is the cross-sectional area of the specimen’s upper surface (cm^2^), *H*_2_ − *H*_1_ represents the difference in the water head and equals 20 mm in the test, and *t* is the time for the test water to flow out (s).

## 3. Experimental Results and Discussion

### 3.1. Compressive Strength

**Figure 8:**
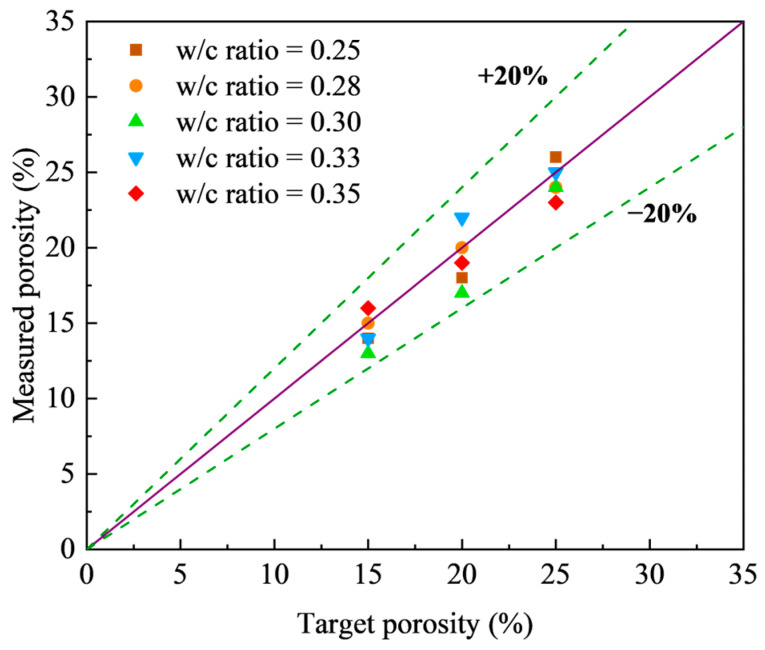
Comparison of measured porosity and target porosity for different w/c ratios.

All experimental results reported in this study are averages derived from three replicate tests. The prepared mixtures demonstrated adequate workability, enabling the consistent production of pervious concrete specimens with homogeneously distributed void structures. Figure 8 illustrates the correlation between the measured porosity and the target porosity across various w/c ratios. This confirms that the measured porosity values were very close to the intended targets, irrespective of the w/c ratio, with an average relative error below 20%.

#### 3.1.1. Effect of Target Porosity on Compressive Strength

Table 7 summarizes the test results of the average elastic modulus for all pervious concrete specimens. As expected, the elastic modulus exhibits a decreasing trend as the target porosity increases. When the w/c ratio equals 0.30, the elastic modulus reaches the maximum regardless of the target porosity. The compressive strengths of pervious concrete were tested at different design porosities (15%, 20%, and 25%) and w/c ratios (0.25, 0.28, 0.30, 0.33, and 0.35). Table 7 presents the experimentally measured compressive strength at different target porosities and w/c ratios. As can be observed, the compressive strength of pervious concrete shows a marked decrease as the design porosity increases. This clearly indicates that the strength of pervious concrete is significantly influenced by its porosity. It is worth noting that this reduction in compressive strength can be linked to both a decrease in the cement dosage and an increase in the aggregate-to-cement ratio. Consequently, this study mitigates the influence of these two factors by employing a smaller gradient in the design porosity.

**Figure 9:**
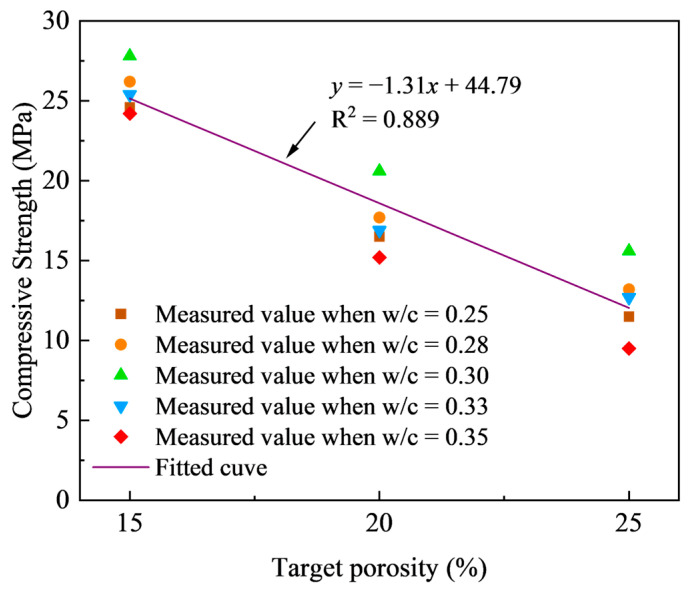
Effect of target porosity on compressive strength for different w/c ratios.

Figure 9 illustrates the impact of target porosity on the compressive strengths of pervious concrete with various w/c ratios. As previously discussed, the compressive strength of pervious concrete shows a linear decrease as porosity increases. Specifically, the average compressive strength decreases from 25.64 MPa to 12.5 MPa as the porosity increases from 15% to 25%. This behavior stems from the reduction in effective bonding interfaces and contact areas between particles with higher porosity, which inherently weakens the material’s load-bearing capacity and reduces cohesion among the granular components. Therefore, an increase in the porosity is associated with a decline in the overall strength of the pervious concrete, regardless of the w/c ratio.

The relationship between the compressive strength and the measured porosity, accounting for different w/c ratios, was effectively modeled using a simple linear reduction equation, which indicates that the compressive strength of pervious concrete reduced linearly with the increase of target porosity.

#### 3.1.2. Effect of w/c Ratio on Compressive Strength

**Figure 10:**
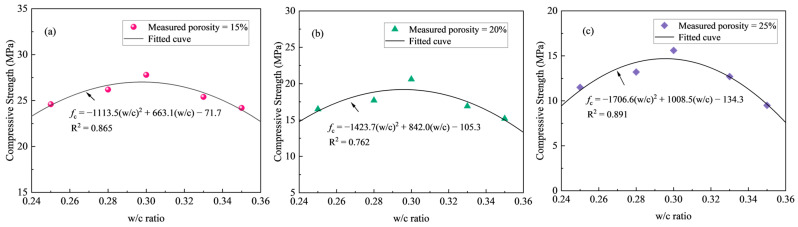
Effect of w/c ratio on compressive strength for measured porosity of (**a**) 15%, (**b**) 20%, and (**c**) 25%.

The variation in the compressive strength of pervious concrete with respect to the w/c ratio is depicted in Figure 10, considering three different porosities. As anticipated, the relationship between the compressive strength and w/c ratio for pervious concrete deviates from that observed in conventional cement concrete. Specifically, reducing the w/c ratio does not lead to a substantial increase in the compressive strength. Furthermore, regardless of the target porosity, the compressive strength of pervious concrete follows a distinct trend: it initially rises and then falls as the w/c ratio increases, peaking at a w/c ratio of 0.30. This behavior can be explained as follows. At a relatively low w/c ratio of 0.25, an insufficient cement hydration results in a weaker binder matrix within the cement paste, consequently reducing the strength of the pervious concrete. When the w/c ratio is as large as 0.35, although full cement hydration may still occur, the binder strength of the cement paste tends to decrease with further increases in the w/c ratio, leading to a decline in the compressive strength of the pervious concrete. At a w/c ratio of 0.30, optimal cement hydration is achieved, maximizing the binder strength of the cement paste and thereby optimizing the mechanical properties of the pervious concrete. This phenomenon aligns with findings reported by Sonebi and Bassuoni [49] and Cui et al. [50].

Based on the test results, regression analysis was employed to establish the relationship between the w/c ratio and compressive strength. Quadratic functions were used to formulate the regression expressions for compressive strength as a function of w/c ratio, which yields:

(4) $$f_{c}=-1113.5{\left(w/c\right)}^{2}+663.1\left(w/c\right)-71.7\;\mathrm{for}\;P=15\%$$

(5) $$f_{c}=-1423.7{\left(w/c\right)}^{2}+842.0\left(w/c\right)-105.3\;\mathrm{for}\;P=20\%$$

(6) $$f_{c}=-1706.6{\left(w/c\right)}^{2}+1008.5\left(w/c\right)-134.3\;\mathrm{for}\;P=25\%$$

From Figure 10, it can be confirmed that the proposed equations can be used to evaluate the relationship between the compressive strength and the w/c ratio of pervious concrete with reasonable accuracy.

#### 3.1.3. Effect of SSA Replacement Ratio on Compressive Strength

Table 8 presents the compressive strengths of SSPC for test specimens with different target porosities and SSA replacement ratios, where the w/c ratio remains constant at 0.3. The results indicate a consistent enhancement in the compressive strength of pervious concrete as the SSA replacement ratio increases, irrespective of the targeted porosity levels. As the porosity increases, the compressive strength exhibits a significant reduction, while the reduction rate of the compressive strength slightly increases with the increment of the SSA replacement ratio. Specifically, the compressive strength decreases by 53.4%, 57.0%, 57.9%, 58.5%, and 58.6% at SSA replacement ratios of 0, 25%, 50%, 75%, and 100%, respectively. Therefore, the maximum compressive strength of 28.4 MPa is attained at a porosity of 15% and a 100% SSA replacement ratio, which means that the coarse aggregate is composed entirely of steel slag. This finding strongly suggests that the improved mechanical properties of the aggregate, resulting from the utilization of SSA, are a critical factor in enhancing the compressive strength of pervious concrete.

**Figure 11:**
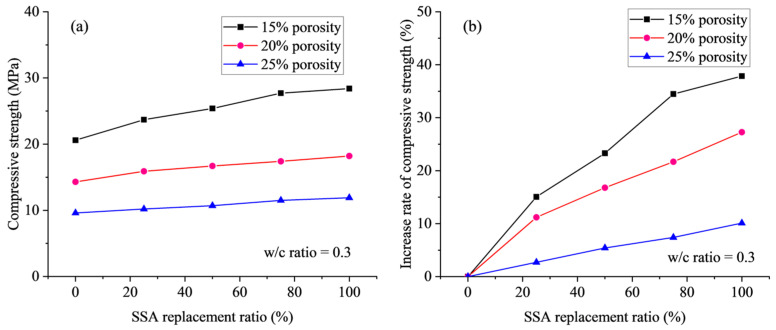
Effect of SSA replacement ratio on (**a**) compressive strength, and (**b**) the increase rate of compressive strength.

The influence of SSA replacement ratio on the measured value and the percentage change in compressive strength across three distinct target porosities (15%, 20%, and 25%) is shown in Figure 11. A notable observation is that the efficacy of the SSA replacement ratio in enhancing the compressive strength gradually diminishes as the porosity increases. Specifically, when the SSA replacement ratio increases from 0 to 100%, the compressive strength shows increases of 37.86%, 22.3%, and 10.1% for porosity levels of 15%, 20%, and 25%, respectively. Therefore, a lower porosity results in a more significant improvement in the compressive strength, and this improvement becomes more significant for a larger SSA replacement ratio. This confirms that the enhancement in aggregate properties through the use of SSA leads to more pronounced strength improvements in pervious concrete with lower porosities, with this enhancement effect diminishing as the target porosity increases.

### 3.2. Permeability Coefficient

#### 3.2.1. Effect of Target Porosity

**Figure 12:**
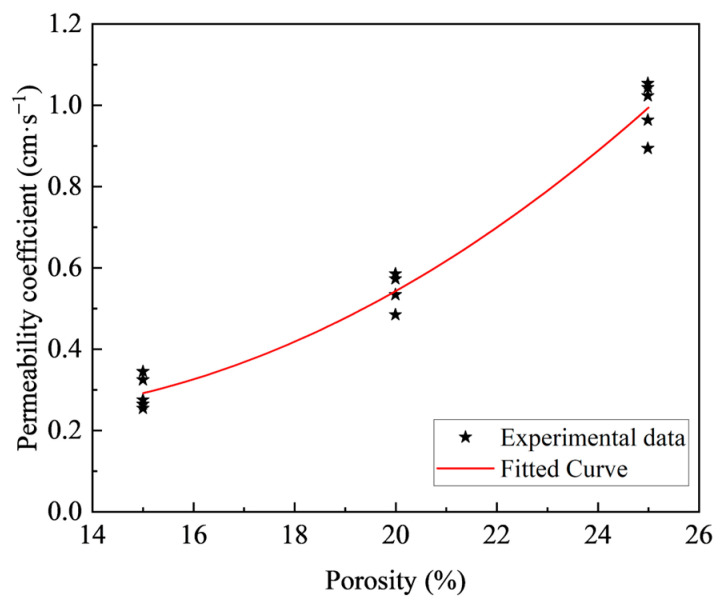
Effect of porosity on the permeability coefficient of pervious concrete.

The test results of the P-wave velocities are provided in Table 7, where it is seen that the P-wave velocity decreases with the increase of the porosity regardless of the w/c ratio. The influence of target porosity on the permeability coefficient, considering various w/c ratios, is presented in Figure 12. A pronounced correlation is observed between the target porosity and the permeability coefficient, a relationship further characterized by an exponential function, while the w/c ratio has no significant influence. As the porosity increases from 15% to 25%, the permeability coefficient exhibits a significant increase from 0.228 to 0.992 cm/s. This behavior can be attributed to the reduced volume of cement paste associated with higher target porosities, which in turn leads to a greater abundance and larger diameter of interconnected pores within the concrete matrix. Accordingly, this altered pore structure diminishes the hydraulic resistance to water flow through the specimen, thereby augmenting the volumetric water flow rate per unit time and finally elevating the permeability coefficient.

#### 3.2.2. Effect of w/c Ratio

**Figure 13:**
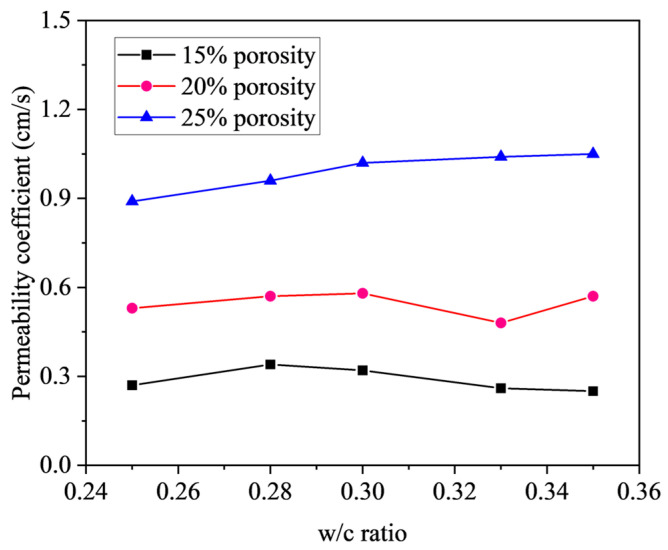
Effect of w/c ratio on permeability coefficient of pervious concrete.

Figure 13 presents the influence of w/c ratio on the permeability coefficient of pervious concrete with different target porosities. It is evident that the influence of the w/c ratio on the permeability coefficient varies significantly with changes in the target porosity. Specifically, when the target porosity is set at 15%, the permeability coefficient peaks at a w/c ratio of 0.28; for a target porosity of 20%, the peak is observed at a w/c ratio of 0.30; and when the target porosity increases to 25%, the pervious concrete with a w/c ratio of 0.35 exhibits the highest permeability. This variation is primarily attributed to the close relationship between the permeability coefficient and the fluidity of the cement paste. At higher fluidity, the paste tends to flow along the pores to the bottom of the concrete, potentially obstructing the pores and reducing the number of interconnected voids, thus decreasing the permeability coefficient. In contrast, at lower fluidity, the cement paste adheres more effectively to the surface of the aggregates, minimizing the impact on pore interconnectivity, thereby preserving a greater number of through pores and enhancing the permeability coefficient. However, the effect of w/c ratio on the permeability coefficient is insignificant for a given target porosity.

#### 3.2.3. Effect of SSA Replacement Ratio

**Figure 14:**
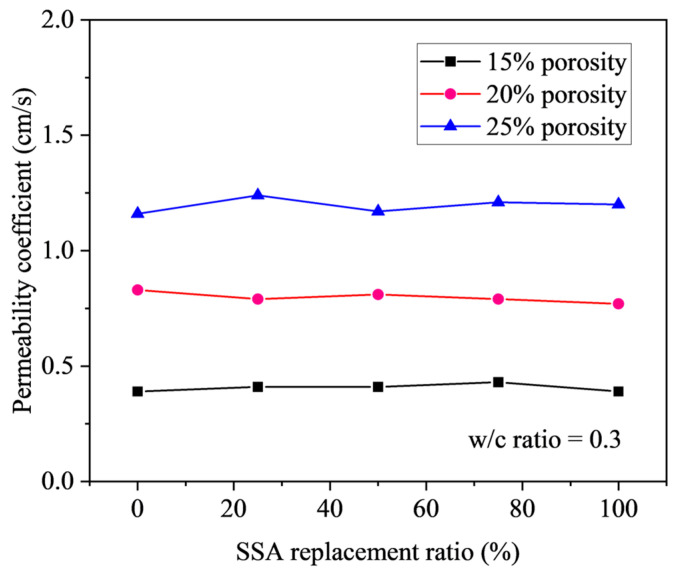
Effect of SSA replacement ratio on permeability coefficient of pervious concrete.

Figure 14 depicts the permeability coefficients of pervious concrete incorporating varying SSA replacement ratios, specifically for target porosities of 15%, 20%, and 25% with a constant w/c ratio of 0.3. The figure reveals a negligible correlation between the permeability coefficient and the SSA replacement ratio. Instead, the permeability coefficient is predominantly governed by the magnitude of the target porosity. For target porosities of 15%, 20%, and 25%, the corresponding permeability coefficients, irrespective of the SSA content, show marginal ranges of 0.39 to 0.43, 0.77 to 0.83, and 1.16 to 1.24 cm/s, respectively. This observation shows that porosity is the principal determinant of the permeability characteristics of pervious concrete.

Theoretically, the minimal influence of the SSA replacement ratio on the permeability coefficient can be rationalized based on the experimental design. In this study, both the steel slag and gravel aggregates possess particle sizes within the 10 to 20 mm range and were substituted on an equal-volume basis. Coupled with a constant w/c ratio of 0.3, this ensured consistent paste fluidity and cohesiveness across all mixtures. Consequently, for a given target porosity, the number of interconnected pores, pore dimensions, and the vertical distribution of the cement paste within the pervious concrete matrix were expected to remain uniform, irrespective of the SSA replacement level. This theoretical uniformity in pore structure and paste distribution consequently leads to comparable permeability coefficients across different SSA replacement ratios.

### 3.3. Relationship Between Porosity, Permeability and Strength

**Figure 15:**
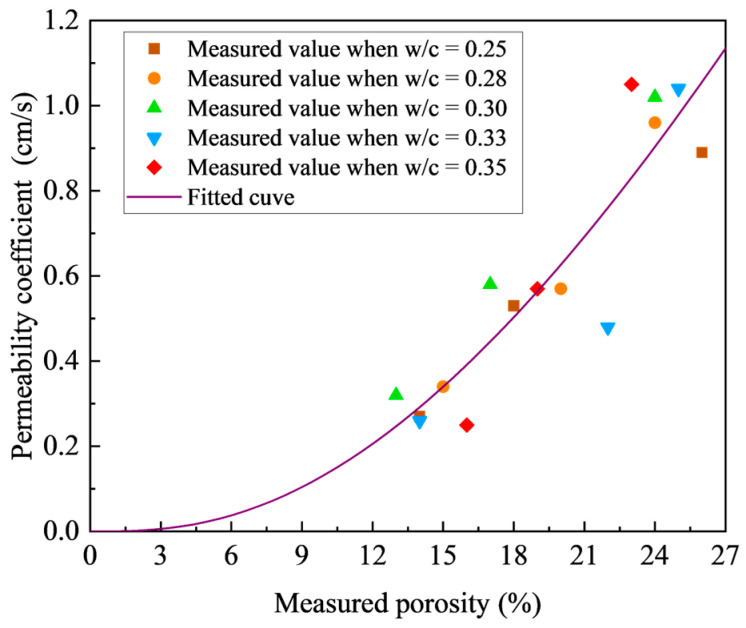
Relationship between porosity and permeability coefficient.

Previous studies have established that for a given porosity, the strength and permeability of pervious concrete represent two critical design factors in practical applications. However, there is limited research quantifying the relationship between porosity, permeability and strength properties, which is crucial for effective mix design and optimization. Figure 15 shows the relationship between permeability and porosity for pervious concrete, demonstrating a clear positive correlation between these parameters. Notably, as porosity increases, the permeability coefficient not only rises but also exhibits an accelerating rate of increase. In contrast, the w/c ratio exhibits a minimal influence on the permeability coefficient, as already demonstrated in Section 3.2. An increase in porosity is typically associated with a reduction in the volume of cement paste. The quantitative relationship between experimentally measured porosity and the permeability coefficient was investigated using various fitting functions.

**Figure 16:**
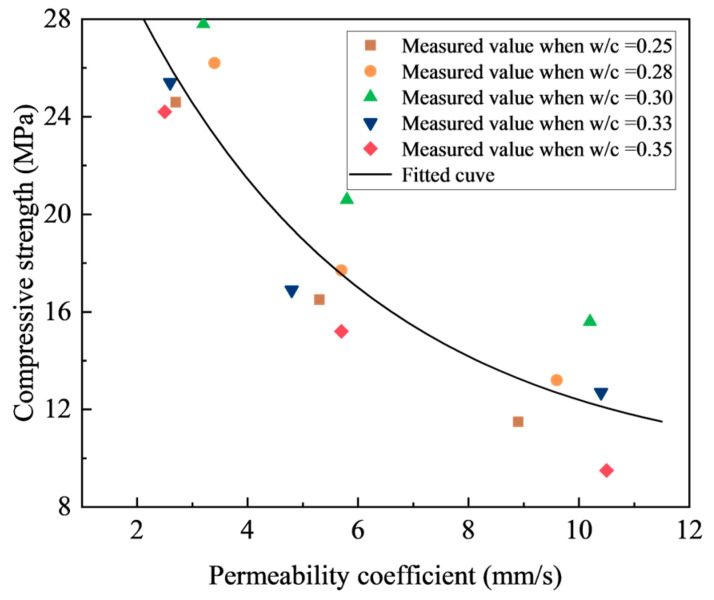
Relationship between permeability coefficient and compressive strength.

As for the relationship between the compressive strength and permeability coefficient, Figure 16 illustrates the trends in the compressive strength as a function of the permeability coefficient, considering different porosities and w/c ratios. A consistent inverse relationship is observed, i.e., increasing the permeability correlates with the declining strength, irrespective of the w/c ratio and porosity. However, the rate of this decline demonstrates a progressively diminishing effect.

This observed strength versus permeability relationship in pervious concrete confirms a negative correlation between the material’s mechanical integrity and its hydraulic conductivity. Consequently, optimizing the balance between strength and permeability requires careful consideration of specific project demands and material characteristics, highlighting the need for tailored design approaches.

The tested solution can be potentially applied in sponge city infrastructure projects requiring balanced mechanical and permeability performance, such as urban pavements (sidewalks, plazas), light-traffic areas, and stormwater management systems. The optimal mix parameters obtained in the present study (i.e., w/c ratio = 0.3, 15% to 25% porosity, and SSA replacement ratio of 100%) provide actionable guidelines for engineers to determine the optimum mix proportion of pervious concrete according to specific engineering requirements. Based on the established empirical relationships between different parameters, the following mix design procedure is proposed for practical application:

(1) Specify the minimum required compressive strength (*f_c_* > *A*) and permeability coefficient (*K* > *B*) based on project-specific engineering requirements and quality control standards.
(2) Utilize the empirical *f_c_* versus *K* relationship established in Figure 16 to derive the “effective interval”. This delimits the desired range for compressive strength [*f_c_* ∈ (A, C)] and permeability coefficient [*K* ∈ (B, D)].
(3) Calculate the required porosity (*P*) corresponding to the mean target permeability coefficient (*K*) using the empirical relationship between *K* and *P* shown in Figure 15.
(4) Identify the optimum w/c ratio that achieves the mean target compressive strength *fc* at porosity *P*, utilizing the empirical relationship between *f_c_* and w/c ratio presented in Figure 10.
(5) Calculate the permeability coefficient (*K*) associated with the optimum w/c ratio using the empirical relationship in Figure 13. Confirm compliance with the target range [*K* ∈ (B, D)].
(6) The preceding steps yield a mix design meeting specified engineering requirements.

## 4. Numerical Simulations

Within the framework of conventional macro-scale modeling, pervious concrete is often idealized as a solid, homogeneous, and isotropic medium. Nevertheless, the predictive accuracy of macro-scale simulations is constrained by their inability to resolve meso-scale phenomena, such as stress distribution and damage-induced cracking processes. Since macro-scale models exhibit limitations in accurately representing the nonlinear mechanical behavior characteristic of the meso-scale during damage and cracking phases [51], this section is dedicated to the development of a meso-scale finite element (FE) model incorporating variable target porosities to enable the prediction of compressive cracking behavior in pervious concrete.

### 4.1. Finite Element Modelling

In the present study, a 2D meso-scale FE model of pervious concrete was developed. Despite the three-dimensional nature of pervious concrete, previous studies have found that the 2D simplified model can be effectively used to predict the stress distributions and damage behavior of pervious concrete [52]. To balance accuracy with computational efficiency, a 2D mesoscopic numerical simulation method was proposed herein, allowing for accurate simulation and analysis of pervious concrete while consuming fewer computing resources.

At the meso-scale, concrete is typically modeled as a three-phase composite consisting of aggregates, the ITZ, and mortar [53]. For static analysis, simplifying assumptions are often made: the coarse aggregates are treated as spherical particles, and the ITZ is represented as a spherical shell surrounding them [54]. In this study, the coarse aggregate size range was set between 10 mm and 20 mm. Pore diameters were categorized into two intervals: 1–2 mm and 2–4 mm. The ITZ thickness was assumed to be 1 mm [55,56]. To accurately calculate the area fraction occupied by coarse aggregates and pores, the Walraven formula, as shown in Equation (7), was applied, through which the calculation results were rounded to yield quantities of coarse aggregates and pores via the plane model area and corresponding formulas [see Equation (8)].

(7) $$p_{c}=p_{k}\left(1.065d^{0.5}d_{\max}^{-0.5}-0.053d^{4}d_{\max}^{-4}-0.012d^{6}d_{\max}^{-6}-0.0045d^{8}d_{\max}^{-8}-0.0025d^{10}d_{\max}^{-10}\right)$$

(8) $$N_{i}=\mathrm{int}\left(A\cdot p_{0}/A_{i}\right)$$

where the parameters are defined as follows: *d* denotes the specific aggregate particle diameter being considered (mm), while *d*_max_ is the maximum aggregate particle size allowed (mm); *p_c_* represents the percentage of aggregate particles smaller than diameter *d*, based on the total aggregate mass (%); *p_k_* is the ratio of the combined volume of coarse and fine aggregates to the total concrete volume, typically assumed to be 75%; *p*_0_ indicates the probability (%) that an aggregate particle falls within a specific size range. In Equation (8), the function int ( ) denotes the rounding function, and *A_i_* is the cross-sectional area occupied by aggregates of a particular particle size within that section.

**Figure 17:**
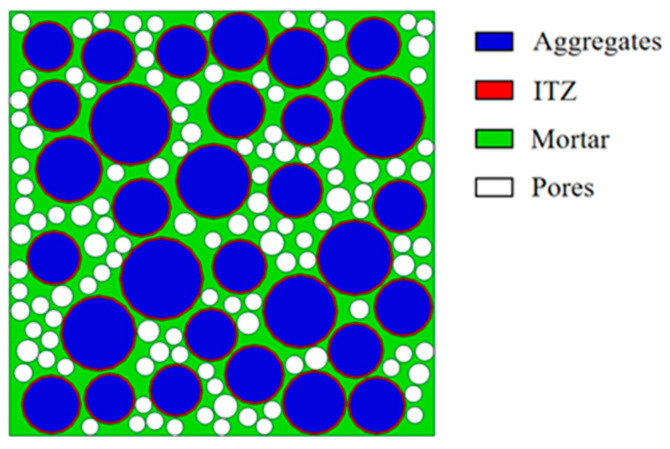
FE model of pervious concrete.

Utilizing the Monte Carlo method the Python programming language (version 3.70), a program was developed to randomly generate and position the 2D aggregates, the interfacial transition zone (ITZ), and pores within the model. The boundaries of each aggregate and pore were designed to ensure no intersection, overlap, or exceedance of the model’s size boundaries. This custom script could then be directly executed within ABAQUS, enabling the efficient creation of the desired aggregate models while avoiding potential compatibility issues associated with programming via external software. Figure 17 illustrates the developed 2D mesoscopic FE model for the cubic pervious concrete specimen, which was formed by combining the aggregates, ITZ, and pores, retaining their intersecting boundaries. For simulation purposes, the CPS3 element type was used, with the model meshed using triangular elements of 2 mm size. The specimens were subjected to uniaxial compressive testing and fixed at the bottom, and an axial pressure load was applied at the top through displacement control at a loading rate of 2 mm/s.

### 4.2. Parameter Calibration

The meso-scale parameters incorporated in the model primarily consist of particle parameters and bonding parameters. The latter category includes aggregate bonding parameters, paste bonding parameters, and ITZ bonding parameters. Particle parameters include density and friction coefficients, while bonding parameters encompass normal stiffness, tangential stiffness, normal bond strength, and tangential bond strength. These mechanical parameters must be calibrated prior to numerical calculations. Since limestone aggregates and reinforced cement paste were used in this study, their intrinsic material properties were similar. To simplify calculations, the same density and effective modulus were assigned to both the aggregate and paste. The model’s normal and tangential bond strengths were calibrated using a trial-and-error approach guided by the actual stress-strain curve of pervious concrete obtained from laboratory tests on pervious concrete with a 15% design porosity. The parameters were iteratively adjusted until the numerical stress-strain curve matched well with the experimental results. The calibrated mesoscale parameters are summarized in Table 9.

### 4.3. Comparison Between Numerical and Experimental Results

**Figure 18:**
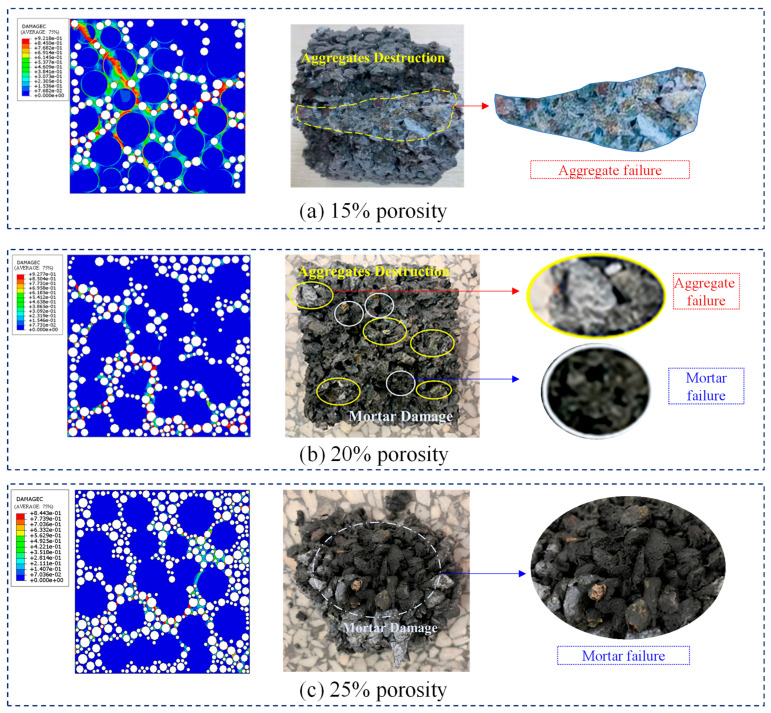
Comparison of predicted and tested failure mode for porosity of (**a**) 15%, (**b**) 20%, and (**c**) 25%.

Figure 18 shows a comparison of the predicted failure modes under compression and the experimental results for three different target porosities. In Figure 18, the notation “e-02” represents “10^−2^”, “e-01” stands for “10^−1^”, and “e+00” means “1”. For the 15% porosity case, the thicker mortar and larger bonding surfaces between aggregates lead to strong resistance in the cementitious material. Thus, failure primarily occurs within the aggregates due to stress concentrations, which agrees with the experimental observations shown in Figure 18a. When the target porosity increases to 20%, both the mortar thickness and the bonding surface between aggregates decrease compared to the 15% porosity case, but are larger than in the 25% porosity case. As a result, the resistance of the cementitious material is intermediate, and the failure mode shows a transition from aggregate failure towards mortar failure between aggregates (see Figure 18b). For the 25% porosity scenario (see Figure 18c), the thinner mortar and smaller bonding surfaces weaken the resistance. Therefore, the mortar between aggregates mainly bears shear or tensile stresses. Failure typically starts in the ITZ and the mortar between closely spaced pores, where interface cracks between mortar and aggregates appear. Once an ITZ element fails, the cracks quickly propagate through the ITZs between adjacent aggregates and mortar. As the damaged mortar regions become continuous, the specimen eventually fails.

**Figure 19:**
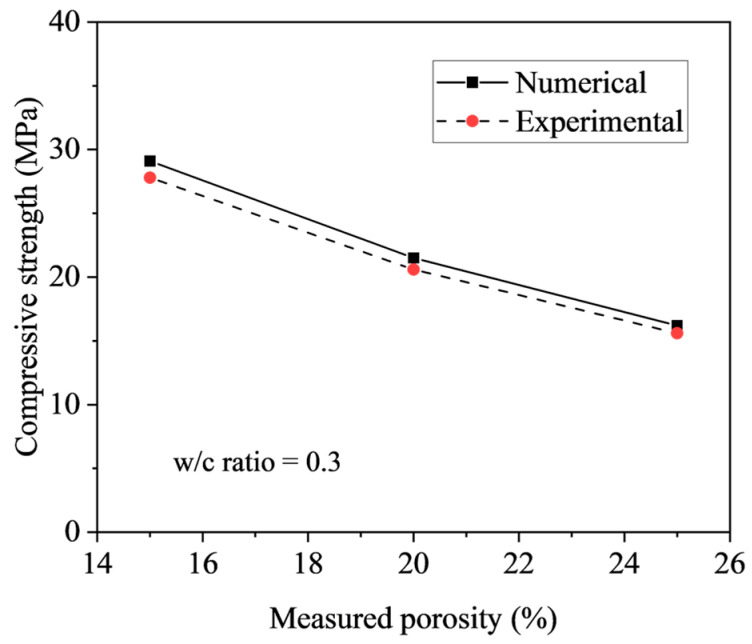
Comparison of predicted and tested compressive strength.

Therefore, it is demonstrated that the predicted failure modes are mainly governed by aggregate penetration for a porosity of 15%, aggregate penetration accompanied by mortar damage for a porosity of 20%, and mortar failure for a porosity of 25%, respectively, which are consistent with the experimental observations. Figure 19 compares the compressive strength of pervious concrete with varying porosities, as predicted by the proposed numerical model, against the corresponding experimental results. It is shown that the average ratio of predicted to experimental load-bearing capacity is 1.04, with a low coefficient of variation (COV) of 0.33%. This close agreement validates the predictive accuracy of the proposed numerical model for assessing the compressive strength and associated failure behavior of pervious concrete across a range of target porosities from 15% to 25%.

It should be pointed out that a main limitation of this study lies in the evaluation of failure modes for PC and SSPC testing. This assessment relies on macroscopic fracture surface images, yielding qualitative descriptions rather than quantitative characterizations. As a result, the effect of the porosity on the compressive strength, permeability, and damage mechanisms of pervious concrete with and without SSA needs to be further investigated based on 2D mesoscale pore characteristics. Integrating advanced image analysis techniques and numerical methods, such as digital image correlation (DIC), would facilitate this objective. Such an approach is expected to enable the precise assessment of the relationship between the predicted results of permeability and compressive strength and the established understanding of the mechanical behavior of pervious concrete.

## 5. Conclusions

This paper has presented an experimental and numerical investigation into the influence of target porosity and w/c ratio on the compressive strength and permeability of pervious concrete. Based on the results obtained, the following conclusions can be drawn:

(1) Pervious concrete cube specimens prepared with an optimal w/c ratio of 0.3 exhibit compressive strengths of 27.8, 20.6, and 15.6 MPa for target porosities of 15%, 20%, and 25%, respectively. Conversely, the permeability coefficients experience a significant increase with porosity, reaching 0.32, 0.58, and 1.02 cm/s for the respective porosity levels.
(2) The porosity shows a significant influence on both the strength and permeability of pervious concrete, while the influence of w/c ratio is marginal. As the porosity increases from 15% to 25%, the permeability coefficient increases considerably from 0.228 to 0.992 cm/s on average, while the average compressive strength decreases from 25.64 MPa to 12.5 MPa.
(3) The incorporation of steel slag aggregate (SSA) leads to an increase in the strength of pervious concrete but has no significant influence on the permeability. For a lower porosity of 15%, pervious concrete with the SSA replacement ratio of 100% exhibits a larger improvement in the compressive strength up to 37.86% compared to conventional pervious concrete.
(4) Empirical models correlating the porosity, strength and permeability of pervious concrete were established and validated through comparison with the experimental results. As the permeability coefficient increases, the compressive strength decreases monotonously while the rate of this reduction diminishes gradually.
(5) A distinct relationship exists between porosity and the failure mechanism of pervious concrete. Lower porosities are associated with a higher propensity for aggregate fracture. As porosity increases from 15% to 25%, the failure mode progressively changes from aggregate penetration to ITZ interface debonding and mortar cracking, leading to strength reductions.

## Figures and Tables

**Table 1:** Coarse aggregates.

Materials	Size (mm)	Apparent Density (kg/m^3^)	Compacted Bulk Density (kg/m^3^)	Clay Content (%)	Needle and Flake Content (%)	Compacted Bulk Porosity (%)	Crush Value (%)
Granite	5–10	2870	1550	0.4	2.2	46	6.4
Steel lag	5–10	3290	1870	-	-	43	6.3

**Table 2:** Chemical components of steel slag.

CaO	Fe_2_O_3_	SiO_2_	Al_2_O_3_	MgO	MnO	P_2_O_3_
37.53	22.32	19.45	6.24	6.04	1.90	1.38

**Table 3:** Main parameters of polycarboxylic acid superplasticizer.

Density (kg/m^3^)	Water Reduction (%)	Solid Content (%)	Alkali Content (%)	Chlorine Ion Content (%)	PH
1049	32	23.64	0.45	0.02	6.0

**Table 4:** Mix proportion of PC.

Concrete Type	No.	Target Porosity (%)	w/c	Cement (kg/m^3^)	Water (kg/m^3^)	Crushed Granite (kg/m^3^)	Additive (%)
PC	PC15_0.25	15	0.25	569	142	1522	0.9
	PC15_0.28	15	0.28	540	157	1522	0.9
	PC15_0.30	15	0.30	522	169	1522	0.8
	PC15_0.33	15	0.33	498	120	1522	0.5
	PC15_0.35	15	0.35	483	132	1522	0
	PC20_0.25	20	0.25	480	120	1522	0.9
	PC20_0.28	20	0.28	456	128	1522	0.9
	PC20_0.30	20	0.30	441	132	1522	0.8
	PC20_0.33	20	0.33	420	139	1522	0.5
	PC20_0.35	20	0.35	408	143	1522	0
	PC25_0.25	25	0.25	391	98	1522	0.9
	PC25_0.28	25	0.28	371	104	1522	0.9
	PC25_0.30	25	0.30	359	107	1522	0.8
	PC25_0.33	25	0.33	342	113	1522	0.5
	PC25_0.35	25	0.35	332	116	1522	0

**Table 5:** Mix proportion of SSPC.

Concrete Type	No.	Target Porosity (%)	SSA Replacement (%)	Steel Lag (kg/m^3^)	Crushed Granite (kg/m^3^)	Cement (kg/m^3^)	Water (kg/m^3^)
SSPC	SSPC15_0	15	0	0	1519	522.0	157
	SSPC15_25	15	25	458	1139	507.8	152
	SSPC15_50	15	50	916	760	507.8	152
	SSPC15_75	15	75	1375	380	507.8	152
	SSPC15_100	15	100	1833	0	475.1	143
	SSPC20_0	20	0	0	1519	441.0	132
	SSPC20_25	20	25	458	1139	426.1	128
	SSPC20_50	20	50	916	760	426.1	128
	SSPC20_75	20	75	1375	380	426.1	128
	SSPC20_100	20	100	1833	0	393.5	118
	SSPC25_0	25	0	0	1519	359.0	107
	SSPC25_25	25	25	458	1139	344.5	103
	SSPC25_50	25	50	916	760	344.5	103
	SSPC25_75	25	75	1375	380	344.5	103
	SSPC25_100	25	100	1833	0	312.5	94

**Table 6:** Mix proportion of coarse aggregates.

Aggregate Type	Apparent Density (kg/m^3^)	Compacted Bulk Density (kg/m^3^)	Compact Packing Porosity (%)	Crush Value (%)
Crushed granite	2870	1550	46	12.6
Granite:SS (3:1)	2890	1590	45	11.2
Granite:SS (1:1)	3110	1710	45	9.5
Granite:SS (1:3)	3220	1770	45	7.8
Steel slag	3290	1870	43	6.4

**Table 7:** Test results of pervious concrete at different target porosities and w/c ratios.

Specimen No.	Target Porosity (%)	Measured Porosity (%)	w/c Ratio	Elastic Modulus (GPa)	Compressive Strength (MPa)	P-Wave Velocity (m/s)	Permeability Coefficient (cm/s)
PC15_0.25	15	14	0.25	24.2	24.6	4505	0.27
PC15_0.28	15	15	0.28	25.0	26.2	4617	0.34
PC15_0.30	15	13	0.30	25.7	27.8	4586	0.32
PC15_0.33	15	14	0.33	24.6	25.4	4493	0.26
PC15_0.35	15	16	0.35	24.0	24.2	4482	0.25
PC20_0.25	20	18	0.25	19.8	16.5	4146	0.53
PC20_0.28	20	20	0.28	20.5	17.7	4179	0.57
PC20_0.30	20	17	0.30	22.1	20.6	4183	0.58
PC20_0.33	20	22	0.33	20.1	16.9	4125	0.48
PC20_0.35	20	19	0.35	19.0	15.2	4178	0.57
PC25_0.25	25	26	0.25	16.5	11.5	3787	0.89
PC25_0.28	25	24	0.28	17.7	13.2	3831	0.96
PC25_0.30	25	24	0.30	19.3	15.6	3883	1.02
PC25_0.33	25	25	0.33	17.4	12.7	3921	1.04
PC25_0.35	25	23	0.35	14.9	9.5	3933	1.05

**Table 8:** Test results of Steel lag pervious concrete at different target porosities.

Specimen No.	Target Porosity (%)	Measured Porosity (%)	SSA Replacement (%)	Compressive Strength (MPa)	Permeability Coefficient (cm/s)
SSPC15_0	15	15	0	20.6	0.39
SSPC15_25	15	14	25	23.7	0.41
SSPC15_50	15	14	50	25.4	0.41
SSPC15_75	15	14	75	27.7	0.43
SSPC15_100	15	16	100	28.4	0.39
SSPC20_0	20	19	0	14.3	0.83
SSPC20_25	20	21	25	15.9	0.79
SSPC20_50	20	20	50	16.7	0.81
SSPC20_75	20	19	75	17.4	0.79
SSPC20_100	20	18	100	18.2	0.77
SSPC25_0	25	25	0	9.6	1.16
SSPC25_25	25	24	25	10.2	1.24
SSPC25_50	25	26	50	10.7	1.17
SSPC25_75	25	27	75	11.5	1.21
SSPC25_100	25	24	100	11.9	1.20

**Table 9:** Calibrated mesoscale parameters of pervious concrete.

Particle Parameters		Linear Parallel Bonding Parameters (Aggregate)			Linear Parallel Bonding Parameters (Paste)		
Density (kg/m^3^)	Friction Factor	Parallel Bond Modulus (GPa)	Normal Bond Strength (MPa)	Shear Bond Strength (MPa)	Parallel Bond Modulus (GPa)	Normal Bond Strength (MPa)	Shear Bond Strength (MPa)
2500	0.7	3.0	11.1	14.0	3.0	9.5	12.0

## Data Availability

The original contributions presented in this study are included in the article. Further inquiries can be directed to the corresponding author(s).
